# *PpMID1* Plays a Role in the Asexual Development and Virulence of *Phytophthora parasitica*

**DOI:** 10.3389/fmicb.2017.00610

**Published:** 2017-04-19

**Authors:** Fang-Yu Hwu, Ming-Wei Lai, Ruey-Fen Liou

**Affiliations:** Department of Plant Pathology and Microbiology, National Taiwan UniversityTaipei, Taiwan

**Keywords:** asexual development, calcium signaling, *MID1*, *Phytophthora*, sporangia

## Abstract

*Phytophthora parasitica* is a notorious oomycete pathogen that causes severe disease in a wide variety of crop species. Infection of plants involves mainly its asexual life stage, including papillate sporangia and biflagellated zoospores, which are the primary dispersal and infection agents of this pathogen. Calcium signaling has been thought as the key regulator for sporangium formation and zoospore differentiation. However, not much is known about the molecular players involved in these processes. In *Saccharomyces cerevisiae*, mating pheromone-induced death 1 (*MID1*) encodes a component of a putative calcium channel. Here, we identified and characterized the function of *PpMID1*, an *MID1* homolog from *P. parasitica*. The expression of *PpMID1* was high in sporangia. Gene silencing of *PpMID1* resulted in the formation of sporangia that lacked papilla and showed a tendency for direct germination. Notably, in response to cold shock to induce zoospore formation, these sporangia showed no sign of cytoplasmic cleavage and thereby failed to form zoospores. Nonetheless, the addition of CaCl_2_ or MgCl_2_ partially recovered the silenced sporangia phenotype, with the formation of papillate sporangia similar to those of the wild type and the release of zoospores upon cold shock. As well, virulence toward *Nicotiana benthamiana* was reduced in the *PpMID1*-silenced transformants. These results indicate a role of *PpMID1* in the asexual development and virulence of *P. parasitica*.

## Introduction

*Phytophthora* belongs to oomycetes that resemble true fungi in morphology and pathogenic lifestyle, yet are phylogenetically distinct from fungi, with brown algae and diatoms as their close relatives (Baldauf et al., 2000). As with other Oomycetes such as *Pythium*, *Phytophthora* can propagate sexually or asexually. Sexual reproduction leads to the formation of thick-walled oospores that may survive harsh environments. In constrast, asexual reproduction involves the formation of sporangia and biflagellated wall-less zoospores.

Sporangia of many *Phytophthora* species may germinate directly through a germ tube or indirectly by releasing zoospores. The released zoospores can swim in water film surrounding soil particles to find potential hosts via chemotaxis. After landing on the plant surface, they encyst rapidly and germinate. Subsequently, germ tubes differentiate to form appressoria and penetration pegs that penetrate into the plant tissue to assist pathogen colonization (Hardham, 2001). Therefore, zoospores play a central role in plant infection and pathogen dispersal and represent a critical component of the disease cycle of most oomycete pathogens.

The formation of sporangia starts with differentiation of mycelia into sporangiophores, which are contiguous and similar in structure to vegetative hyphae (Hemmes, 1983). Subsequently, nuclear division occurs rapidly within the sporangiophores, followed by a quick flow of nuclei and cytoplasm into terminal swellings, which then develop into sporangia (Christen and Hohl, 1972; Maltese et al., 1995). Sporangium formation may be triggered by changes in environmental factors such as nutrients, light, humidity, and pH (Erwin and Ribeiro, 1996). In some *Phytophthora* spp., sporangia can form spontaneously as cultures age, whereas others require depletion of media nutrients to ensure mass production of sporangia (Erwin and Ribeiro, 1996). Completion of sporangia formation is marked by the formation of a basal plug on the bottom to delimit the multinucleate sporangium from the coenocytic hyphae and an apical papilla on the top, which is involved in sporangium germination and zoospore release (Maltese et al., 1995).

The formation of zoospores can be triggered by cool temperatures, a process known as “cold shock,” which induces cytokinesis of the sporangial cytoplasm and subsequent compartment of a single nucleus into each zoospore (Ribeiro, 1983). Zoosporogenesis is accompanied by changes in cytoskeletal organization and vesicle distribution as well as movement of ions such as calcium (Hyde et al., 1991; Jackson and Hardham, 1996). In *Phytophthora cinnamomi*, cold shock elicited an instant increase in the concentration of calcium in the sporangial cytoplasm, which was followed by a second increase of calcium concentration during cytoplasmic cleavage to regulate cytokinesis (Jackson and Hardham, 1996). In *P. infestans*, zoosporogenesis was impaired by treatment with the calcium channel blocker verapamil, the calmodulin antagonist trifluoperazine, and the inositol trisphosphate (IP3)-receptor-gated calcium channel antagonist 2-aminoethoxydiphenyl borate (Judelson and Roberts, 2002; Tani et al., 2004). These results suggest an important role for calcium signaling in zoosporogenesis of oomycetes.

Recent studies further demonstrated that zoosporogenesis involves major changes in expression profiles of many genes including those encoding putative protein kinases, transcription factors, ion channels and other regulator proteins, which are regulated via multiple signaling pathways (Kim and Judelson, 2003; Tani et al., 2004; Judelson et al., 2008, 2009). In addition, regulatory proteins that participate in sporangium formation and zoospore release of *P. infestans* include a protein kinase (Judelson and Roberts, 2002), the G-protein beta subunit (Latijnhouwers and Govers, 2003), the NIF transcriptional regulators (Judelson and Tani, 2007), and PiGK4, a G-protein-coupled receptor with a phosphatidylinositol phosphate kinase domain (Hua et al., 2013). Nonetheless, not much is known about the molecular players involved in calcium signaling in the asexual reproduction of *Phytophthora*.

**FIGURE 1:**
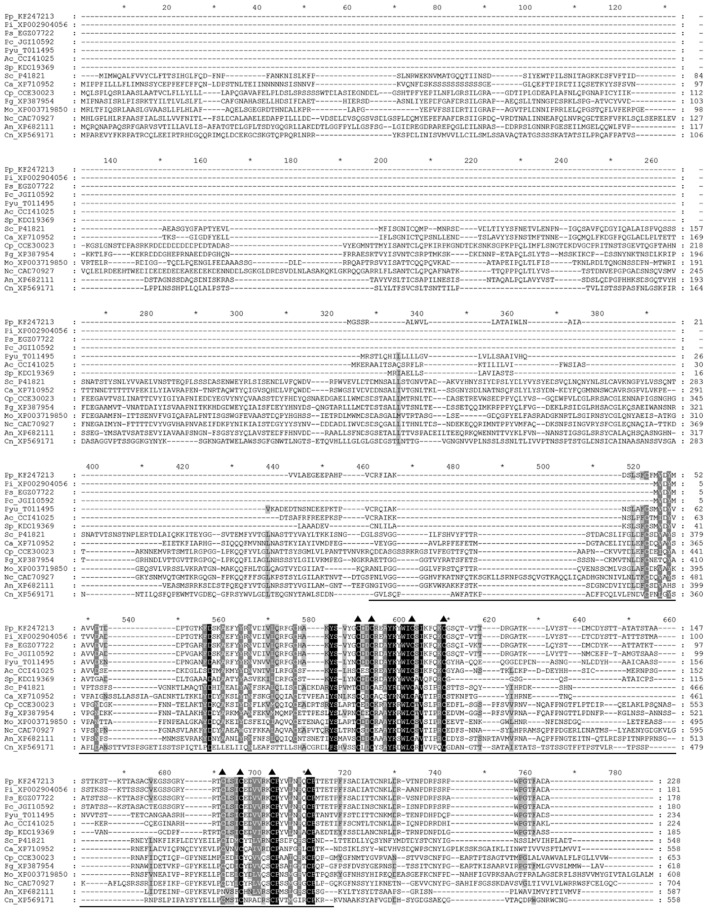
**Amino acid sequence alignment of PpMID1 and its homologs from other organisms.** Amino acid sequences were aligned by using Clustal X 1.83. Triangles mark positions of the conserved cysteine residues and the underline indicates the location of the MID1 domain. Names of the sequences are shown with the first two letters representing the organisms, followed by GenBank accession numbers or JGI protein IDs. Ac, *Albugo candida*; An, *Aspergillus nidulans*; Ca, *Candida albican*; Cn, *Cryptococcus neoformans*; Cp, *Claviceps purpurea*; Fg, *Fusarium graminearum*; Mo, *Magnaporthe oryzae*; Nc, *Neurospora crassa*; Pc, *Phytophthora capsici*; Pi, *P. infestans*; Pp, *P. parasitica*; Ps, *P. sojae*; Pyu, *Pythium ultimum*; Sc, *Saccharomyces cerevisiae*; Sp, *Saprolegnia parasitica.*

Calcium signaling through the Ca^2+^-binding protein calmodulin and the Ca^2+^-calmodulin-dependent phosphatase calcineurin is an important pathway in many organisms including fungi, acting to modulate the process of budding, mating, stress response, and virulence (Thewes, 2014). In fungi, cellular calcium levels are regulated by multiple channels and transporters, with the low-affinity calcium uptake system (LACS) and high-affinity calcium uptake system (HACS) as two major calcium uptake pathways (Locke et al., 2000; Muller et al., 2001). When calcium availability is high, the primary calcium entry route is LACS, which is minimally composed of mating factor-induced gene 1 (FIG1), a transmembrane calcium channel and regulatory protein (Muller et al., 2003). In contrast, during low calcium availability, the major calcium entry route is HACS, which is composed of calcium channel homolog 1 (CCH1), mating pheromone-induced death 1 (MID1; Locke et al., 2000), and the PMP22_Claudin superfamily member Ecm7 (Martin et al., 2011; Ding et al., 2013). *CCH1* encodes a transmembrane protein homologous to the α1-subunit of the L-type voltage-gated calcium channel in mammals (Fischer et al., 1997; Locke et al., 2000). In contrast, *MID1* shows no overall sequence similarity to any known β-subunits of the L-type voltage-gated calcium channel (Iida et al., 1994). In *Saccharomyces cerevisiae*, deletion mutants of *CCH1* or *MID1* in a *MAT*a background died shortly after exposure to α-factor in a calcium-limited medium (Iida et al., 1994; Fischer et al., 1997). An *Aspergillus nidulans* mutant (*midA*) carrying a *MID1* mutation had highly branched hyphae and showed at the hyphal apex an aberrant distribution pattern of the Spitzenkörper, the organizing center for hyphal growth and morphogenesis (Wang et al., 2012). As well, the CCH1-MID1 channel is indispensable for the survival of fungal pathogens such as *Candida albicans* and *Cryptococcus neoformans* in low-calcium environments (Locke et al., 2000; Bonilla et al., 2002; Liu et al., 2006), and also function to maintain calcium homeostasis in response to environmental and endoplasmic reticulum stress (Hong et al., 2010).

*Phytophthora parasitica* Dastur (= *Phytophthora nicotianae* Breda de Haan) causes root rot, foot rot, leaf blight, and fruit rot in a wide variety of crop species (Erwin and Ribeiro, 1996). During asexual reproduction, it produces terminally on the sporangiophores papillate, non-caducous, spherical to obturbinate sporangia, which may release zoospores after incubation at cool temperature. In this study, we demonstrated that the expression of *PpMID1*, an *MID1* homolog of *P. parasitica*, is highly induced in *P. parasitica* sporangia and silencing *PpMID1* causes severe defects in the asexual reproduction of the pathogen, including the formation of aberrant sporangia and inability to produce zoospores, as well as a reduction in virulence. These results indicate an important role for *PpMID1* in the asexual development and virulence of *P. parasitica*.

## Materials and Methods

### Growth of *P. parasitica* and Plant Material

*Phytophthora parasitica* isolates (731, 991, and 94069) were provided by P. J. Ann (Taiwan Agricultural Research Institute, WuFeng, Taiwan). For routine culture, they were grown on 20% V8 juice agar (20% Campbell’s V8 juice, 0.02% CaCO_3_, and 2% agar) at 25°C in the dark. *Nicotiana benthamiana* was grown in a mixture of peat moss, perlite, and vermiculite at 25°C under a 12-h light/dark regime.

### Molecular Cloning and Sequence Analysis of *PpMID1*

The genomic and cDNA sequences of *PpMID1* were amplified by PCR and reverse transcriptase PCR, respectively, with the primers Ppmid1_795F and Ppmid1_821R (Supplementary Table S1) and cloned into pGEM-T Easy vector (Promega, Fitchburg, WI, USA). Following sequence analysis, conserved protein domains were predicted by use of the InterProScan 5 server^1^. Alignment of *PpMID1* and its homologs from other organisms involved use of Clustal X. Phylogenetic trees were generated by the neighbor-joining algorithm implemented in MEGA 6.06 with the default parameters. Nodal support of the tree was estimated by bootstrapping with 1,000 pseudoreplicate data sets.

### Isolation of RNA

Total RNA from different life stages of *P. parasitica*, including mycelia, sporangia, zoospores, cysts, and germinating cysts were prepared with use of TriZol reagent (Invitrogen-Life Technologies, Carlsbad, CA, USA) as described by Yan and Liou (2006). RNA from inoculated plants was isolated by using the Plant Total RNA Extraction Kit (Viogene-BioTek, New Taipei City, Taiwan).

### Pathogen Inoculation

For inoculation, the *P. parasitica* isolate was grown in 20% V8 juice agar for 5 days. Then, mycelial disks (5 mm id.) were excised from the margin and used to inoculate detached 4th or 5th leaves of 5-week-old *N. benthamiana* plants. The inoculated leaves were kept in a plastic box with high humidity at 25°C in the dark.

### Quantitative RT-PCR

Residual DNA present in the RNA solution was removed by use of the Turbo-DNA *free* kit (Ambion, Huntingdon, UK). First-strand cDNA was synthesized by using SuperScript III reverse transcriptase (Invitrogen-Life Technologies), with 1 μg RNA as the template and 25 μM VdT (V: A, C, or G at the 3′) as the primer. Quantitative PCR involved the Step One Plus Real-Time PCR System (Applied Biosystems, Foster City, CA, USA). The reaction mixture (20 μL) contained 10 μL Power SYBR Green PCR Master Mix (Applied Biosystems), 0.5 μM primers (Supplementary Table S1), and 1 μL cDNA product diluted 10 times. The settings for PCR were 95°C/10 min, 42 cycles of 95°C/15 s, 60°C/1 min, followed by melting curve analysis. For analysis of gene expression in *P. parasitica*, the level of WS21, which encodes a ribosomal protein, was an internal control for normalization (Yan and Liou, 2006).

### Construction of the Silencing Vector

With pHAM34 (a gift from Dr. Y. C. Wang, Department of Plant Pathology, Nanjing Agricultural University, Nanjing, China) as the template, a DNA fragment containing 5′HAM34 and 3′HAM34 (Judelson and Michelmore, 1991) was amplified by using pro_ham34_HindIII_F.2/ter_ham34_XbaI_R as primers and subcloned into the *Hin*dIII and *Xba*I sites of pTH209 (a gift from Dr. H. Judelson, Department of Plant Pathology and Microbiology, University of California, Riverside, CA) to generate pEX (Supplementary Figure S1). Subsequently, an intron sequence of the tomato NIPRa gene (Liou et al., unpublished data) was amplified by using primers pHP_intron3_F and pHP_intron3_R (Supplementary Table S1), with the addition of three different restriction sites on either end, namely *Nhe*I, *Avr*II, and *Sac*II sites at the 5′ end and *Sac*I, *Spe*I, and *Bam*HI sites at the 3′ end. This DNA fragment was then inserted into the *Sma*I site of pEX to generate pHP. For construction of pHPdest, two Gateway destination cassettes provided by the Gateway vector conversion system (Invitrogen-Life Technologies) were ligated into pHP in an opposite orientation, accomplished by use of *Nhe*I and *Sac*II sites at the 5′ end and *Sac*I and *Bam*HI sites on the 3′ end of the intron sequence. To construct the silencing vector, the partial sequence of *PpMID1* was amplified with the primers PhyMID1_F and PhyMID1_R (Supplementary Table S1) and subcloned into pENTR/D-TOPO vector (Invitrogen-Life Technologies). LR recombination reaction then involved use of pHPdest and the Gateway LR clonase II enzyme mix (Invitrogen-Life Technologies) to obtain the silencing vector pHPdest::*PpMID1*.

### Preparation of Protoplasts and Polyethylene Glycerol (PEG)-mediated Transformation

Protoplasts of *P. parasitica* were prepared by using young mycelial mats as described (McLeod et al., 2008), followed by transformation of the protoplasts with some modifications. Three days after growth of *P. parasitica* in 20% V8 juice medium (20% Campbell’s V8 juice and 0.02% CaCO_3_), the mycelia were harvested by filtration and washed in 0.4 M mannitol to weed out residual medium. The mycelia (counted as one volume) were digested at 25°C for 45 min in 14 volumes of a digestion solution [0.5% (w/v) cellulase (Sigma-Aldrich), 1.5% (w/v) lysing enzyme (L1412, Sigma-Aldrich), 0.4 M mannitol, 20 mM KCl, 10 mM CaCl_2_, and 20 mM MES, pH 5.7]. Then the digested solution was filtrated through 4 layers of Miracloth (EMD Millipore, Billerica, MA, USA), and protoplasts were collected by centrifugation for 3 min with 1,000 g at 25°C. After a rinse with 25 mL W5 solution (154 mM NaCl, 125 mM CaCl_2_, 5 mM KCl, and 2 mM MES, pH 5.7), protoplasts were resuspended in 5 mL W5 solution and incubated on ice for 30 min, then collected by centrifugation at 1000 *g* for 3 min, followed by resuspension in the MMg solution (0.4 M mannitol, 15 mM MgCl_2_, and 4 mM MES, pH 5.7) to a final concentration of 1 × 10^6^ protoplasts/mL and incubation at room temperature for 10 min. For PEG-mediated transformation, 1-mL aliquots of the protoplast suspension were mixed with 30–50 μg of plasmid DNA in 15-mL Falcon tubes, then incubated on ice for 10 min. Subsequently, 580 μL of PEG 4000 solution [40% (v/v) PEG 4000 (Sigma-Aldrich), 0.2 M mannitol, and 0.1 M CaCl_2_] was added slowly to each Falcon tube and the tube was gently inverted several times to mix the contents thoroughly. This process was repeated twice at an interval of 30 s to add a total of 1.74 mL PEG 4000 solution. The mixture was then incubated on ice for 20 min, followed by the addition of 2 mL V8-M medium (20% V8 juice, 0.5 M mannitol, and 10 mM CaCl_2_) and 2 min later 8 mL V8-M medium. Finally, the mixture was incubated at 25°C for 7 h to allow protoplasts to regenerate, with the addition of 50 ppm ampicillin to prevent bacterial contamination.

After regeneration, protoplasts were examined under a microscope for their recovery. Once the regenerated protoplasts began to germinate, the cells were suspended in 20% V8 juice medium and plated on 20% V8 agar plates that contained 10 ppm geneticin. After incubation at 25°C in the dark for 4 days, colonies appearing on the agar plate were transferred to fresh 20% V8 agar plates amended with 10 ppm geneticin for a second round of selection. Only transformants passing at least two rounds of geneticin selection underwent further analysis by PCR primed with nptF1 and transR2, which target the backbone of the silencing vector.

### Recovery of Silenced Sporangia

To induce sporangium formation, mycelial blocks of *P. parasitica* (2 mm×2 mm) excised from a 7-day-old culture were immersed in 1% V8 juice medium (a 20x dilution of the 20% V8 juice medium) alone or amended with the test solution: 50 mM CaCl_2_, 50 mM MgCl_2_, or 150 mM mannitol. The osmotic pressure of each solution was adjusted to the same level according to the van’t Hoff law (π = *iCRT*). After incubation at 25°C under white light for 3 days, the mycelial blocks were examined under a microscope for the formation of sporangia.

### Staining of Sporangia with DAPI and FM 4–64

Sporangia were first stained with DAPI (4′, 6-diamidino-2-phenylindole; Invitrogen-Life Technologies), then FM 4–64 (Invitrogen-Life Technologies) as described (Zhang et al., 2012) with some modifications. Briefly, sporulating mycelial blocks were incubated in a DAPI solution [300 nM in Hank’s balanced salt solution (HBSS)] for 10 min and the residual DAPI was washed out with HBSS. Then FM 4–64 solution (5 μg/mL in HBSS) was added, followed by immediate examination of sporangia under a Leica DMLB microscope (Buffalo Grove, IL) equipped with filter cube A (BP 340–380 nm, LP 425 nm; for DAPI) or I3 (BP 450–490 nm, LP 515 nm; for FM 4–64). Images were captured by using a Canon (Ohta-ku, Tokyo, Japan) digital camera EOS 550D.

### The Mating Experiment

Mycelia blocks (5 mm × 5 mm) of test isolates of *P. parasitica* were placed pairwise on the glass slides inside a Petri dish. After incubation at 25°C in the dark for 7 days, the morphology of oospores was examined and their number was counted under a microscope.

### Trypan Blue Staining

Trypan blue staining was performed as described (Wilson and Coffey, 1980). *N. benthamiana* leaves were boiled in 96% ethanol and then for 45 s in a trypan blue solution prepared by mixing two volumes of 96% ethanol with one volume of a solution containing 0.03% (w/v) trypan blue (Fluka, Buchs, Switzerland) in lactophenol (lactic acid:phenol:glycerol:ddH_2_O = 1:1:1:1). The stained leaves were then rinsed several times with 96% ethanol until the background was clear.

### Statistical Analysis

Statistically significant differences were analyzed by Fisher’s least significant difference test. Significance was set at P < 0.05 and determined by use of PASW Statistics 18 (SPSS Inc, Chicago, IL, USA).

## Results

### Sequence Analysis of *PpMID1*

To identify *P. parasitica* gene(s) homologous to *MID1*, we performed a BLAST search of the *P. parasitica* (INRA-310) genome database of the Broad Institute, with *S. cerevisiae MID1* (GAA25818.1) used as a query and found a homolog, PPTG_18661 [*P. parasitica INRA-310* (V2)], that contains an intron and encodes a protein of 228 amino acid residues (aa) with 26% identity with yeast *MID1*. We then cloned the genomic and cDNA sequence of this gene, hereafter named *PpMID1* (GenBank accession no. KF247213), and found the sequences identical to that of PPTG_18661. InterProScan analysis revealed the presence of an N-terminal signal peptide (aa 1–25) as well as two signature matches: aa 35–195 for IPR024338 (stretch-activated cation channel MID1) (**Figure 1**) and aa 48–120 for IPR029604 (transmembrane protein FAM155, functionally uncharacterized). Notably, PpMID1 is much smaller than fungal MID1, with conservation confined to the carboxyl terminus, especially the 9 cysteine residues at specific positions (**Figure 1**, triangles), which are essential for the function of MID1 in *S. cerevisiae* (Maruoka et al., 2002). Homologs of *MID1* were identified in the genome databases of other oomycetes, including *P. capsici*, *P. infestans*, *P. sojae*, *Pythium ultimum*, *Albugo candida*, and *Saprolegnia parasitica.* Phylogenetic analysis indicated that the *MID1* homologs from *Phytophthora* spp. formed a cluster with the other three oomycete pathogens but distinct from *MID1* homologs from true fungi (Supplementary Figure S2).

### Expression of *PpMID1* Is Highly Induced in Sporangia and *In Planta*

**FIGURE 2:**
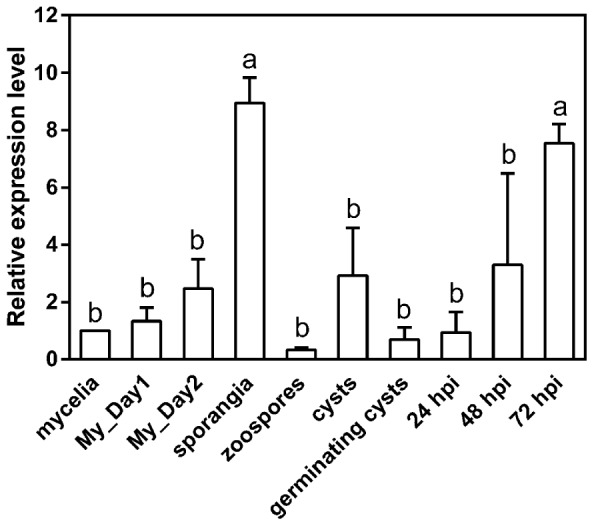
**Expression of *PpMID1* is highly induced in the sporangia of *Phytophthora parasitica* and *in planta*.** qRT-PCR of total RNA from different life stages of *P. parasitica* and 5-week-old *Nicotiana benthamiana* leaves inoculated with *P. parasitica*. Raw data were normalized to the transcript level of *WS21* as an internal control and shown as fold change relative to that of mycelium. Data are mean ± SD from 3 independent experiments. Different letters indicate significant difference by Fisher’s least significant difference test (*P* < 0.05). “My_Day1” and “My_Day2” indicate mycelia harvested at 1 and 2 days, respectively, after light treatment for inducing sporangium formation. hpi: hours post-inoculation.

To examine the expression pattern of *PpMID1*, we prepared total RNA from different life stages of *P. parasitica* for quantitative RT-PCR analysis. The data were normalized to transcript levels of *WS21* as an internal control and expressed as fold change relative to the level in mycelium. *WS21* encodes a ribosomal protein and shows constant expression throughout different life stages of *P. parasitica* (Yan and Liou, 2006). When the mycelia were subjected to light treatment to induce sporangium formation, the expression of *PpMID1* increased gradually from 1 to 2 days after light treatment (**Figure 2**, My_Day1 and My_Day2), with a peak for sporangia collected at 3 days after light treatment (**Figure 2**). We also analyzed the expression of *PpMID1* in *N. benthamiana* leaves inoculated with *P. parasitica*. Compared to the transcript level detected in germinating cysts, *PpMID1* was slightly induced at 48 h post-inoculation (hpi) but highly induced at 72 hpi, when abundant sporangia formed on the infected leaves (**Figure 2**). These results suggest an important role of *PpMID1* during the formation of sporangia in *P. parasitica*.

### Generation of *PpMID1*-silenced Transformants

**FIGURE 3:**
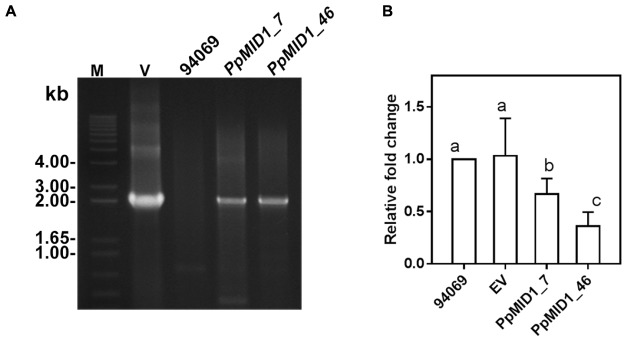
**Analyses based on PCR and qRT-PCR show both *PpMID1_7* and *PpMID1_46* as *Phytophthora parasitica* transformants. (A)** PCR analysis. Genomic DNAs extracted from the *PpMID1-*silenced transformants (*PpMID1_7* and *PpMID1_46*) and wild-type *P. parasitica* (94069) were amplified with the primers nptF1 and transR2. V, plasmid DNA as the PCR template; M, size marker (in kb). **(B)** The expression of *PpMID1* is downregulated in the mycelium of *PpMID1-*silenced transformants. qRT-PCR analysis of total RNA from the mycelia of *PpMID1_7* and *PpMID1_46*, the empty vector transformant (EV), and the wild type (94069). Raw data were normalized by transcripts of *WS21* as an internal control and shown as fold change relative to that of the wild-type strain. Data are mean ± SD from 3 independent experiments. Different letters indicate significant difference by Fisher’s least significant difference test (*P* < 0.05).

To characterize the function of *PpMID1*, we generated *PpMID1-*silenced transformants by double-stranded RNA-mediated gene silencing with pHPdest::*PpMID1* used as the silencing vector (Supplementary Figure S1). pHPdest was constructed by using pTH209 (Judelson et al., 1991) as the backbone to allow generation of the silencing vectors by use of the Gateway cloning system. The strategy for constructing pHPdest and the silencing vector is detailed in Materials and methods. After transformation of *P. parasitica* protoplasts with the silencing vector pHPdest::*PpMID1* and regeneration, we obtained a total of 540 putative transformants based on geneticin resistance. Most of them died after several rounds of subculture on V8 medium amended with geneticin. The remaining 48 isolates were further examined for changes in phenotypes, especially formation of sporangia and their characteristics, and analyzed by PCR primed with nptF1 and transR2 (Supplementary Table S1). Nonetheless, 46 isolates, likely transient transformants, showed geneticin resistance for around 6 months and were unable to grow in the V8 medium amended with geneticin in the subsequent subculture. Only *PpMID1_7* and *PpMID1_46* turned out to be stable transformants. Meanwhile, we obtained several *P. parasitica* transformants carrying the empty pHPdest vector as a control. Both *PpMID1_7* and *PpMID1_46* showed generated amplicons of the expected size (**Figure 3A**) and significantly lower expression of *PpMID1* in the mycelia than the wild-type *P. parasitica* and the empty vector transformants (EV) as revealed by qRT-PCR (**Figure 3B**). Expression of *PpMID1* in the sporangia was not analyzed because of the small number of sporangia produced by the silenced transformants.

### *PpMID1*-silenced Transformants Show Defects in Mycelia Morphology and Produce Abnormal Sporangia

**FIGURE 4:**
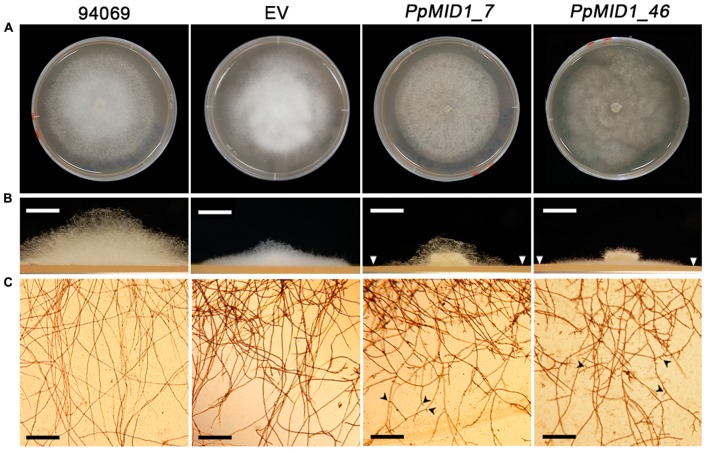
***PpMID1-*silenced transformants produce less aerial mycelia and mycelia in liquid culture are highly branched.** **(A)** Colony morphology. Photographs were taken 7 days after subculture. **(B)** *PpMID1-*silenced transformants produced less aerial mycelia on solid medium. Mycelia disks (5 mm id.) of silenced transformants (*PpMID1_7* and *PpMID1_46*), EV, and wild-type *Phytophthora parasitica* (94069) were transferred to 20% V8 juice agar and grown in the dark at 25°C. Photographs for the side view of each colony were taken 3 days after subculture. Arrowheads, colony margins of silenced transformants. Bar = 5 mm. **(C)** Mycelia produced by *PpMID1-*silenced transformants in liquid medium are highly branched. Mycelia disks (5 mm id.) of *PpMID1_7*, *PpMID1_46*, EV, and the wild type (94069) were transferred to 20% V8 juice medium and grown at 25°C in the dark. Photographs were taken under a microscope 1 day later. Arrowheads, branched hyphae. Bar = 100 μm.

To investigate the role of *PpMID1* in different life stages of *P. parasitica*, we examined the growth rate and morphology of mycelium in the *PpMID1-*silenced transformants *PpMID1_7* and *PpMID1_46*. When grown in 20% V8 juice agar at 25°C for 3 days, the growth of *PpMID1_7* was similar to that of the wild-type strain (94069) and EV control, as revealed by colony diameters (**Table 1**). Interestingly, *PpMID1_7* and *PpMID1_46* produced much less aerial mycelia than the wild type and EV at 3 and 7 days after subculture (**Figures 4A,B**). Young hyphae produced by *PpMID1_7* and *PpMID1_46* tended to branch more frequently than did the wild type and EV (**Figure 4C**), with an average branch-to-branch interval of 148.9 ± 24.9 and 114.0 ± 25.7 μm for *PpMID1_7* and *PpMID1_46*, respectively, as compared with 422.2 ± 46.2 μm for the wild type and 365.2 ± 48.9 μm for EV (**Table 1**). As well, branches of hyphae produced by *PpMID1*-silenced transformants were relatively short (**Figure 4C**), probably due to impeded hyphal growth after branching. These results suggest the involvement of *PpMID1* in hyphal branching and tip growth.

**Table 1:** *PpMID1-*silenced transformants produce less sporangia and branch more frequently.

*Phytophthora parasitica*	Growth rate (mm/d)^1,5^	No. of sporangia^2,5^	No. of zoospores^3,5^	Branch-to-branch hyphal interval (μm)^4,5^
94069	14.2 ± 1.4^a^	967 ± 53^a^	755 ± 79^a^	422.2 ± 46.2^a^
EV	15.4 ± 0.1^a^	878 ± 72^a^	873 ± 60^a^	365.2 ± 48.9^a^
*PpMID1_7*	12.8 ± 2.5^a^	446 ± 31^b^	0 ± 0^b^	148.9 ± 24.9^b^
*PpMID1_46*	10.9 ± 1.1^a^	600 ± 93^b^	6 ± 1^b^	114.0 ± 25.7^b^


*^1^Mycelia disks (5 mm id.) of *PpMID1-*silenced transformants (*PpMID1_7* and *PpMID1_46*), the empty vector transformant (EV), and the wild-type strain of *P. parasitica* (94069) were transferred to 20% V8 juice agar plates and grown in the dark at 25°C. Diameter of the colonies was measured daily for three consecutive days. ^2^After induction for sporangium formation, the number of sporangia present in three 20% V8 juice agar blocks (2 mm × 2 mm) were counted. ^3^After induction of zoospore formation by cold shock (19°C, 30 min) and release of zoospores from sporangia, the numbers of zoospores present in 10 μL induction solution were determined. ^4^*P. parasitica* was grown in the same way as described in ‘1’. For better observation, mycelia disks (5 mm id.) excised at day 5 were immersed in 60 μL 20% V8 juice medium and pictures were taken under a microscope 3 days later. For each isolate, six branch intervals were measured by using ImageJ. ^5^Data are mean ± SD from 3 independent experiments; means followed by different letters indicate significant difference by Fisher’s least significant difference test (*P* < 0.05).*

**FIGURE 5:**
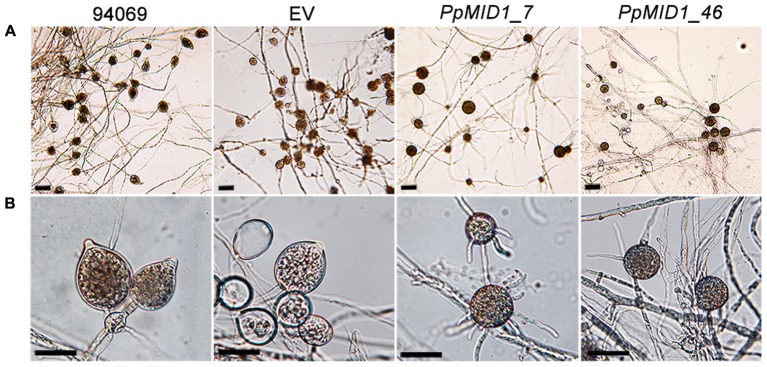
**Sporangia produced by *PpMID1-*silenced transformants show aberrant morphology.** Mycelial blocks of *PpMID1_7* and *PpMID1_46*, EV, and wild-type *Phytophthora parasitica* (94069) were incubated in 1% V8 juice medium under light at 25°C to induce sporangium formation. After 3 days, photographs were taken under a microscope. **(A)** Formation of sporangia; **(B)** Four-fold magnification of **(A)**. Bar = 25 μm.

To determine whether *PpMID1* is involved in the formation of sporangia, we examined the number and morphology of sporangia produced by the transformants. The wild-type *P. parasitica* produced papillate, ovoid, and vesiculogen-containing sporangia, from 20 to 25 μm in diameter (**Figure 5**), with a mean of 967 ± 53 per 2-mm–square agar block. The EV control produced a similar amount of sporangia, 878 ± 72 per 2-mm-square agar block (**Table 1**), which were papillate, vesiculogen-containing, and slightly more rounded. Sporangia from other 5 empty-vector transformants also displayed similar characteristics. In contrast, the mean number of sporangia for *PpMID1_7* and *PpMID1_46* was 446 ± 31 and 600 ± 93 per 2-mm–square agar block (**Table 1**), respectively, significantly less than the wild type and EV. Moreover, all these sporangia were non-papillate and round-shaped, from 10 to 25 μm in diameter, and lacked vesiculogen (**Figure 5**). When cold-shocked to induce zoospore formation, sporangia of the wild type and EV released a mean of 755 ± 79 and 873 ± 60 zoospores per 10 μL induction solution, respectively. In contrast, sporangia of *PpMID1_7* were unable to release any zoospores. Instead, they tended to germinate directly, with 3 to 15 germ tubes sprouting from the round-shaped sporangia (**Figure 5**). Most sporangia produced by *PpMID1_46* germinated directly, producing 1 to 3 germ tubes, and some produced a very small number of zoospores (**Table 1**), which were usually two to three times larger than normal ones. Thus, *PpMID1* silencing interfered with the process of not only sporangium formation but also zoospore production.

### *PpMID1*-silenced Transformants Show Defects in Cytoplasmic Cleavage of Sporangia

**FIGURE 6:**
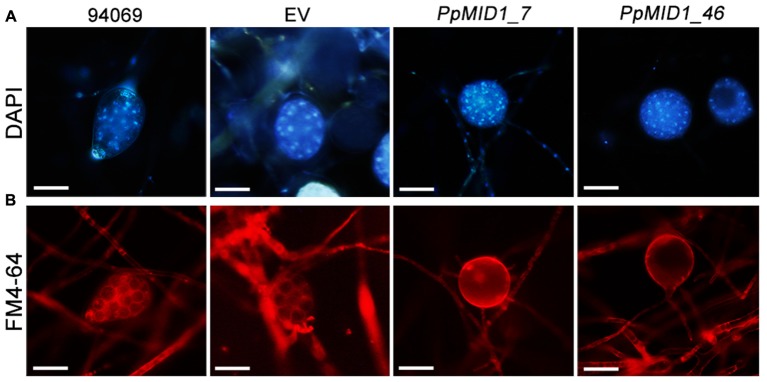
**Sporangia of *PpMID1-*silenced transformants show defective cytoplasmic cleavage.** Mycelia blocks of *PpMID1_7* and *PpMID1_46*, EV, and wild-type *Phytophthora parasitica* (94069) were incubated in 1% V8 juice medium under light at 25°C to induce sporangium formation. After 3 days, the sporangia were cold-shocked at 19°C for 30 min to trigger zoospore differentiation, then stained with: **(A)** DAPI for nuclear division; **(B)** lipophilic dye FM 4–64 to examine the occurrence of cytoplasmic cleavage. Bar = 20 μm.

To determine whether the failure to release zoospores results from a defect in zoospore differentiation or zoospore release, we stained sporangia with DAPI and the lipophilic dye FM 4–64 after cold shock. DAPI staining revealed the presence of multiple nuclei in sporangia of the wild type and EV, which distributed evenly in the sporangial cytoplasm (**Figure 6**). Moreover, staining with FM 4–64 for plasma membrane demonstrated the occurrence of sporangial cytoplasmic cleavage in response to cold shock (**Figure 6**). The silenced sporangia showed multiple nuclei after DAPI staining but no sign of cytoplasmic cleavage on cold shock (**Figure 6**). Hence, *PpMID1* silencing impaired the process of cytoplasmic cleavage. As a result, silenced sporangia could not produce zoospores.

### Addition of CaCl_2_ or MgCl_2_ Recovers the Morphology of Sporangia in *PpMID1*-silenced Transformants and Reclaims Their Ability to Produce Zoospores

**FIGURE 7:**
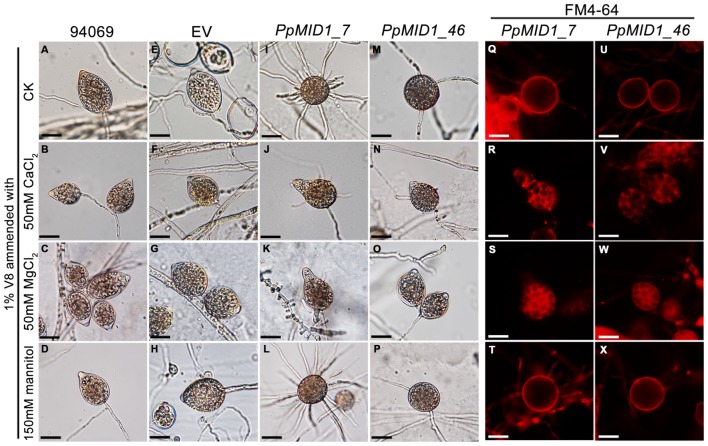
**Addition of CaCl_2_ or MgCl_2_ restores the defective sporangia in *PpMID1-*silenced transformants.** Mycelial blocks of *PpMID1_7* and *PpMID1_46*, EV, and wild-type *Phytophthora parasitica* (94069) were incubated in 1% V8 juice medium with the indicated reagents under light at 25°C for 3 days to induce sporangium formation. As a control, sporangia were induced in 1% V8 juice medium without any amendment (ck). The sporangia were then subjected to cold shock at 19°C for 30 min and stained with FM 4–64 to examine cytoplasmic cleavage. **(A–P)**: Morphology of sporangia produced by the wild type (94069; **A–D**), EV **(E–H)**, *PpMID1_7* **(I–L)**, and *PpMID1_46* **(M–P)**. **(Q–X)**: cytoplasmic cleavage in sporangia produced by *PpMID1_7* **(Q–T)** and *PpMID1_46* **(U–X)**. Bar = 20 μm.

*PpMID1* is predicted to encode a subunit of the high-affinity Ca^2+^ channel. *PpMID1* silencing likely disturbs the influx of calcium ions to impair the process of sporangium formation and cytoplasmic cleavage. To test this hypothesis, mycelia blocks of silenced transformants were incubated in 1% V8 juice medium amended with 50 mM CaCl_2_ under light to induce sporangium formation. As controls, similar experiments were performed by replacing CaCl_2_ with 50 mM MgCl_2_ (an alternative cation) or 150 mM mannitol (a non-ionic osmolite). Moreover, to avoid the effect caused by a difference in osmolarity, each medium was adjusted to the same osmotic pressure according to the van’t Hoff law. The wild type and EV produced papillate, ovoid sporangia with CaCl_2_, MgCl_2_, or mannitol in the medium (**Figures 7A–H**). Remarkably, the addition of CaCl_2_ or MgCl_2_ reversed the morphology of some sporangia produced by *PpMID1_*7 and *PpMID1_*46 (**Table 2**). These sporangia became papillate, ovoid, and bore vesiculogen, similar to sporangia produced by the wild type (**Figures 7J,K,N,O**). However, the papillae of sporangia produced by *PpMID1_*7 were slightly enlarged and irregular-shaped (**Figures 7J,K**). In contrast, with mannitol amendment, the silenced sporangia remained unaltered (**Table 2** and **Figures 7L,P**), similar to those produced in the absence of any amendment (**Figures 7I,M**). Further staining with FM 4–64 revealed rescued cytoplasmic cleavage in silenced sporangia with the addition of CaCl_2_ or MgCl_2_ (**Figures 7R,S,V,W**), but not mannitol (**Figures 7T,X**) and the control without any amendment (**Figures 7Q,U**).

**Table 2:** Morphology of sporangia produced by *PpMID1-*silenced transformants is reversed with the addition of calcium or magnesium ions.

	Percentage of recovered sporangia in 1% V8 juice medium containing^1^		
	
Transformant	50 mM CaCl_2_	50 mM MgCl_2_	150 mM mannitol
*PpMID1_7*	14.8 ± 5.1	32.5 ± 3.9	0.3 ± 0.4
*PpMID1_46*	24.1 ± 2.5	20.6 ± 6.0	2.0 ± 0.5


*^1^Mycelia blocks (2 mm × 2 mm) of the *PpMID1-*silenced transformants (*PpMID1_7* and *PpMID1_46*) were incubated in 1% V8 juice medium amended with indicated ingredients. Following induction of sporangium formation, the number of all sporangia formed in three mycelia blocks was counted. Sporangia bearing a papilla were considered “recovered in morphology” and shown as percentage of the total number of sporangia. Data are mean ± SD from 3 independent experiments.*

### *PpMID1*-silencing Has No Effect on Oospore Formation

**FIGURE 8:**
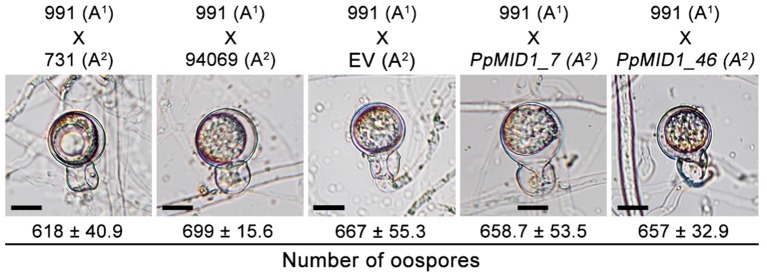
**Silencing of *PpMID1* has no effect on oospore formation.** Pairwise testing of *PpMID1_7*, *PpMID1_46*, EV, and wild-type strains of *Phytophthora parasitica* (94069, 991, and 731) for their ability to mate and produce oospores. Each paired agar culture was incubated at 25°C in the dark for 7 days, then examined under a microscope for oospore formation. Numbers under each photograph indicate the mean ± SD number of oospores present in 5 × 5 mm^2^ agar blocks from 3 independent experiments (*n* = 3). Bar = 12.5 μm.

*MID1* plays a key role in the mating process of *S. cerevisiae* (Iida et al., 1994). To determine whether *PpMID1* is also involved in the sexual reproduction of *P. parasitica*, we performed mating experiments and counted the number of resultant oospores. The wild-type strain (94069) used for generating the silenced strains has the A2 mating type. On mating *PpMID1_7* or *PpMID_46* with a *P. parasitica* isolate of the A1 mating type (isolate number 991), the formation of oospores was similar both in number and morphology to those formed on mating of the wild type (94069) or EV with the A1 strain (**Figure 8**). Therefore, *PpMID1* silencing had no effect on the mating and oospore formation of *P. parasitica*.

### *PpMID1*-silenced Transformants Show Reduced Virulence and Abnormal Features in Infected Plants

**FIGURE 9:**
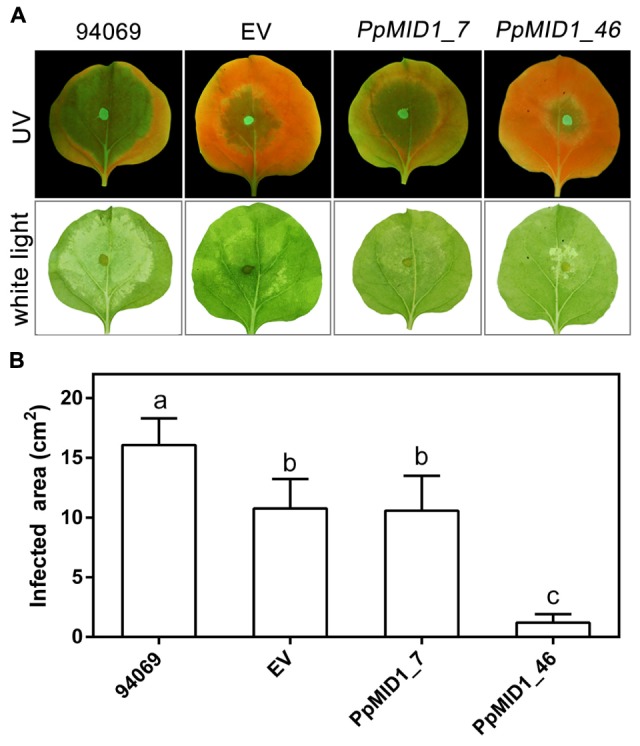
***PpMID1-*silenced transformants show reduced virulence on plants. (A)** Disease symptoms on *Nicotiana benthamiana* leaves caused by *Phytophthora parasitica* infection. For inoculation, mycelia disks (5 mm id.) of *PpMID1_7* and *PpMID1_46*, EV, or wild-type *P. parasitica* (94069) were transferred to the center of leaves from 5-week-old *N. benthamiana*. After incubation in a moisture box at 25°C for 72 h, disease symptoms were examined and photographed under ultraviolet light (UV) or white light. **(B)** Quantification of infected areas on the inoculated *N. benthamiana* leaves. Infected areas on the inoculated leaves shown in **(A)** under UV light were measured by using ImageJ. Data are mean ± SD from 4 independent experiments (each with six leaves). Different letters indicate significant difference by Fisher’s least significant difference test (*P* < 0.05).

**FIGURE 10:**
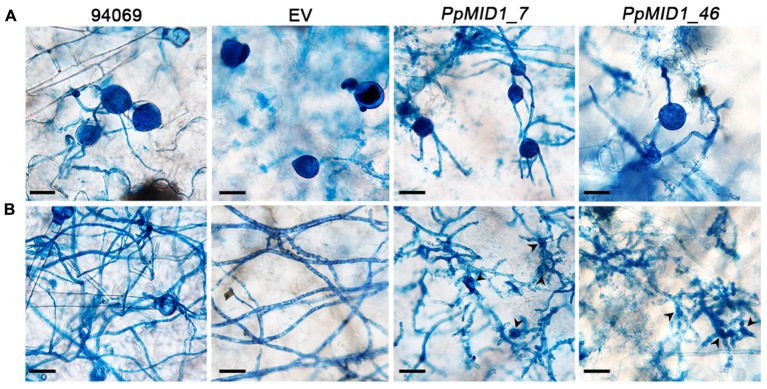
***PpMID1-*silenced transformants generate highly branched mycelia and aberrant sporangia.** Mycelia disks (5 mm id.) of *PpMID1_7*, *PpMID1_46* or wild-type *Phytophthora parasitica* (94069) were transferred to the center of leaves from 5-week-old *Nicotiana benthamiana*. After incubation in a moisture box at 25°C for 72 h, inoculated leaves were stained with trypan blue and photographed under a microscope. **(A)** Silenced transformants produced sporangia that germinated directly. Bar = 25 μm. **(B)** Silenced transformants tended to form deformed and highly branched mycelia. Arrowheads, bulged mycelia of the silenced transformants. Bar = 25 μm.

To determine whether downregulation of *PpMID1* affects the virulence of *P. parasitica*, we inoculated detached leaves of 5-week-old *N. benthamiana* with mycelia disks of the *PpMID1-*silenced transformants. When inoculated with the wild-type *P. parasitica*, the leaves showed water-soaking symptom that covered approximately half of the leaf area by 48 hpi. The lesion then enlarged to encompass almost the whole leaf by 72 hpi (**Figure 9A**). In contrast, in the leaves inoculated with either silenced strain, the disease symptom was alleviated (**Figure 9A**), with a significant difference between infected areas caused by the wild type and silenced transformants (**Figure 9B**), although the EV showed reduced virulence as well. To discover why *PpMID1* silencing reduced the virulence of *P. parasitica*, we stained infected *N. benthamiana* leaves with trypan blue. Microscopy examination revealed the presence of abundant sporangia on *N. benthamiana* leaves infected by the wild type at 72 hpi (**Figure 10A**), accompanied by the presence of some zoospores nearby. In contrast, sporangia were barely seen on leaves infected with *PpMID1_7* or *PpMID1_46*. Of the 10 leaves examined, only one contained sporangia that germinated directly. Moreover, branched hyphae often bulged in the middle or ends (**Figure 10B**, arrowheads). Thus, *PpMID1* silencing affects the virulence of *P. parasitica*, likely caused by a failure in normal hyphal branching and sporangium formation.

## Discussion

Calcium signaling is involved in the regulation of asexual reproduction in *P. parasitica*, which encompasses sporangium formation and zoospore differentiation (von Broembsen and Deacon, 1997; Warburton and Deacon, 1998). Despite previous efforts to elucidate the underlying mechanism, knowledge about the relevant signaling components has been limited, especially those involved in calcium transportation. Here, we demonstrate that *PpMID1* is essential for the asexual reproduction of this pathogen.

*PpMID1* is a homolog of *MID1* that together with *CCH1* forms the only high-affinity calcium channel in the plasma membrane of fungal cells (Liu et al., 2006). *S. cerevisiae MID1* contains an ORF of 548 aa and alone is capable of forming a calcium-permeable, cation-selective stretch-activated channel in Chinese hamster ovary cells (Kanzaki et al., 1999). In contrast, *MID1* of *C. neoformans* with 558 aa does not show any independent channel activity under calcium depletion conditions (Hong et al., 2010). The predicted amino acid sequence and size of *PpMID1* are similar to its homologs from other Oomycetes. However, these proteins are conserved only to the C-terminal regions of fungal *MID1*, especially the highly conserved cysteine residues (**Figure 1**). In *S. cerevisiae*, these cysteine residues are essential for the function of *MID1* (Maruoka et al., 2002). As well, they are likely involved in the formation of MID1 oligomeric structures that are associated with CCH1 (Locke et al., 2000; Hong et al., 2010, 2013). In this regard it is interesting to note that, despite the identification of *MID1* homologs in *P. parasitica* and other oomycetes, no *CCH1* homolog is found in oomycetes according to the information obtained from FungiDB (Stajich et al., 2012). Further work is required to determine whether *PpMID1* shows independent channel activity similar to yeast *MID1*.

Silencing of *PpMID1* had no effect on the growth rate of *P. parasitica* (**Table 1**). However, it did cause defective hyphal branching and aerial hypha formation, likely due to disturbed calcium homeostasis in the mycelia of *P. parasitica*. Of note, intracellular calcium concentrations regulate hyphal extension, orientation and branching in various fungi. Low calcium level caused enhanced hyphal branching in *Fusarium graminearum* and *N. crassa* (Dicker and Turian, 1990; Robson et al., 1991a,b), and chelation of extracellular calcium or deletion of *MID1* and *CCH1* disturbed thigmotropism and severely reduced curve formation in *C. albicans* (Brand et al., 2007, 2009). A recent study demonstrated highly branched hyphae of an *A. nidulans MID1* mutant (*midA*) showing at the hyphal apex an aberrant distribution pattern of the Spitzenkörper (Wang et al., 2012). Nonetheless, not much is known about the regulation of hyphal branching in oomycetes. Additional studies are needed to determine the mechanism that underlies regulation of hyphal branching by calcium in these organisms.

In response to cold shock, sporangia of *Phytophthora* spp. undergo cytoplasmic cleavage, followed by the formation and release of zoospores. Our *PpMID1*-silenced transformants produced fewer sporangia with abnormal morphology: round-shaped and lacking papilla. Moreover, sporangia tended to germinate directly or, owing to failure of cytoplasmic cleavage as revealed by FM 4–64 staining, formed none or only a very small number of zoospores. In *P. cinnamomi*, a transient increase in calcium concentration in the sporangial cytoplasm was required for the initiation of cytoplasmic cleavage in sporangia (Jackson and Hardham, 1996). As well, treatments to interfere with calcium homeostasis disturbed sporangia germination and zoospore release in *P. infestans* (Hill et al., 1998; Judelson and Roberts, 2002). The failure of our *PpMID1*-silenced transformants to produce normal sporangia and to release zoospores was likely caused by reduced expression of *PpMID1* and thereby interference in calcium influx. Studies of various fungal species indicated that calcium supplementation could rescue the aberrant phenotypes of *MID1* mutants in various fungi (Iida et al., 1994; Cavinder et al., 2011; Wang et al., 2012; Harren and Tudzynski, 2013). In our study, the addition of CaCl_2_ substantially reversed the morphology of silenced sporangia and recovered cytoplasmic cleavage in response to cold shock, which supports an essential role of *PpMID1* in calcium signaling. Not all sporangia recovering cleavage was likely due to differential silencing effects of *PpMID1*. Intriguingly, the addition of MgCl_2_ could also reverse the morphology and recover cytoplasmic cleavage in the silenced sporangia. In concert with our results, magnesium could substitute for calcium to rescue the suppression of sporangial cleavage and zoospore release imposed by calcium chelators in *P. infestans* (Hill et al., 1998). Magnesium is one of the most abundant divalent cations in cells, with its cellular content tightly regulated. RNA profile analysis performed in *S. cerevisiae* demonstrated a close and complicated relationship between magnesium and calcium homeostasis (Wiesenberger et al., 2007). Moreover, magnesium may bind calmodulin and other EF-hand proteins, thereby modulating their calcium binding activity (Grabarek, 2011). Nonetheless, these studies pointed to the possibility that an excess of magnesium may attenuate calcium signaling. Further study is required to define the possible alternative pathway(s) adopted by *PpMID1*-silenced transformants to restore the aberrant phenotypes with the addition of MgCl_2_.

In *S. cerevisiae*, *MID1* mutants die on receiving the opposite mating hormone, which indicates the important role of the molecule in the mating process of yeast (Iida et al., 1994). In *Fusarium graminearum* (sexual-stage *Gibberella zeae*), the agent causing head blight in wheat and barley, deletion of *MID1* resulted in the production of predominately abnormal ascospores and a sharp reduction in ascospore discharge activity (Cavinder et al., 2011). However, *MID1* mutants of *N. crassa* could mate and produce viable ascospores (Lew et al., 2008). Similarly, silencing *PpMID1* had no effect on the mating process of *P. parasitica*. Therefore, the role of *MID1* in sexual reproduction may vary depending on the organism.

In summary, reduced expression of *PpMID1* can cause severe defects in hyphal branching, sporangium formation, and cytoplasmic cleavage in sporangia of *P. parasitica*, which supports an important role of *PpMID1* in asexual reproduction in this species. The inability to produce normal hyphae and sporangia can also reduce the virulence of the silenced transformants. Further effort is required to elucidate how *PpMID1* is involved in the modulation of calcium homeostasis in this pathogen.

## Author Contributions

Conceived and designed the experiments: FYH, MWL, and RFL. Contributed to reagents/materials/analysis tools: RFL. Performed the experiments: FYH and MWL. Analyzed the data: FYH, MWL, and RFL. Wrote the manuscript: FYH, MWL, and RFL.

## Conflict of Interest Statement

The authors declare that the research was conducted in the absence of any commercial or financial relationships that could be construed as a potential conflict of interest.

## Footnotes

1 http://www.ebi.ac.uk/Tools/pfa/iprscan5/
