# Supplementary material for: PpMID1 Plays a Role in the Asexual Development and Virulence of Phytophthora parasitica
Source: Front Microbiol. 2017 Apr 19;8:610. doi: 10.3389/fmicb.2017.00610 (PMC5395580; doi:10.3389/fmicb.2017.00610)
Supplement: Supplementary file 1 [file Table_1.DOC]

**Table S1. A list of primers used in this study**

| **Name** | **Sequence (5’ to 3’)** | **Target** |
| --- | --- | --- |
| Ppmid1_795F | CGTGTCTCGATATGATGTTCTCTTCAAAAT | *PpMID1* |
| Ppmid1_821R | TTGTTCCGTGTCTCGATATGATGTTC |  |
| Ppmid1_q94F | TGCTTCATGGTGGATTACATGG | *PpMID1* |
| Ppmid1_q94R | CCACGTTGCGGTAGTAAAATTC |  |
| PhyMID1_F | CACCCACGCCAAGTACAGCGTCTACGG | *PpMID1* |
| PhyMID1_R | CATTTCCTCACGACATCTTCGCAGAT |  |
| pHP_intron3_F | GGATCCACTAGTGAGCTCACTTACCAAAAAATTAC | Intron 3 of NPA100 |
| pHP_intron3_R | GCTAGCCCTAGGCCGCGGGTTTCAAACACAACAC |  |
| pro_ham34_HindIII_F.2 | GTTCAAGCTTCCTCTGATGGACAAAGGGTCGCCTC | 5’Ham34 |
| ter_ham34_XbaI_R | GGTTACATGTTTGCCATTGTTATGGTTGGT | 3’Ham34 |
| nptF1 | GGAGAGGCTATTCGGCTATGA | Silencing vector |
| transR2 | GATGACGGTGAAAACCTCTGAC |  |
| WS21_F1 | CTCCAGAACGTGTACATCCG | WS21 |
| WS21_R1 | TAGCGCCCTTCTCCTCAG |  |
