# Supplementary figures and images for: PpMID1 Plays a Role in the Asexual Development and Virulence of Phytophthora parasitica
Source: Front Microbiol. 2017 Apr 19;8:610. doi: 10.3389/fmicb.2017.00610 (PMC5395580; doi:10.3389/fmicb.2017.00610)

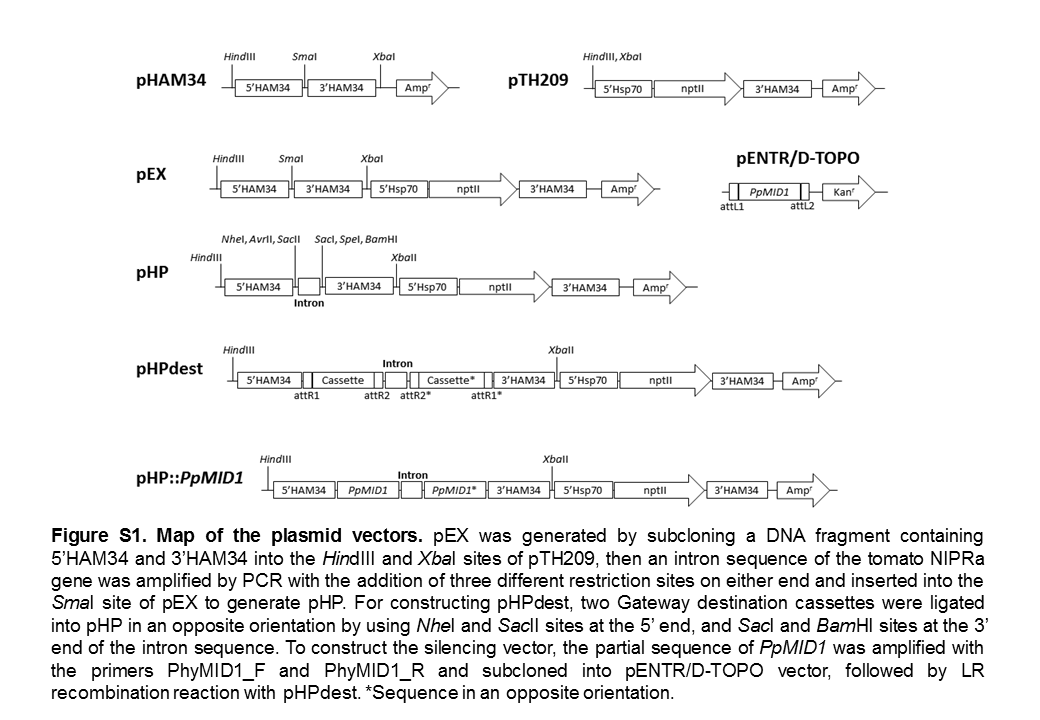

Supplement: Supplementary file 2 [file Image_1.TIF]
