# Supplementary material for: PpMID1 Plays a Role in the Asexual Development and Virulence of Phytophthora parasitica
Source: Front Microbiol. 2017 Apr 19;8:610. doi: 10.3389/fmicb.2017.00610 (PMC5395580; doi:10.3389/fmicb.2017.00610)
Supplement: Supplementary file 3 [file Image_2.pdf]

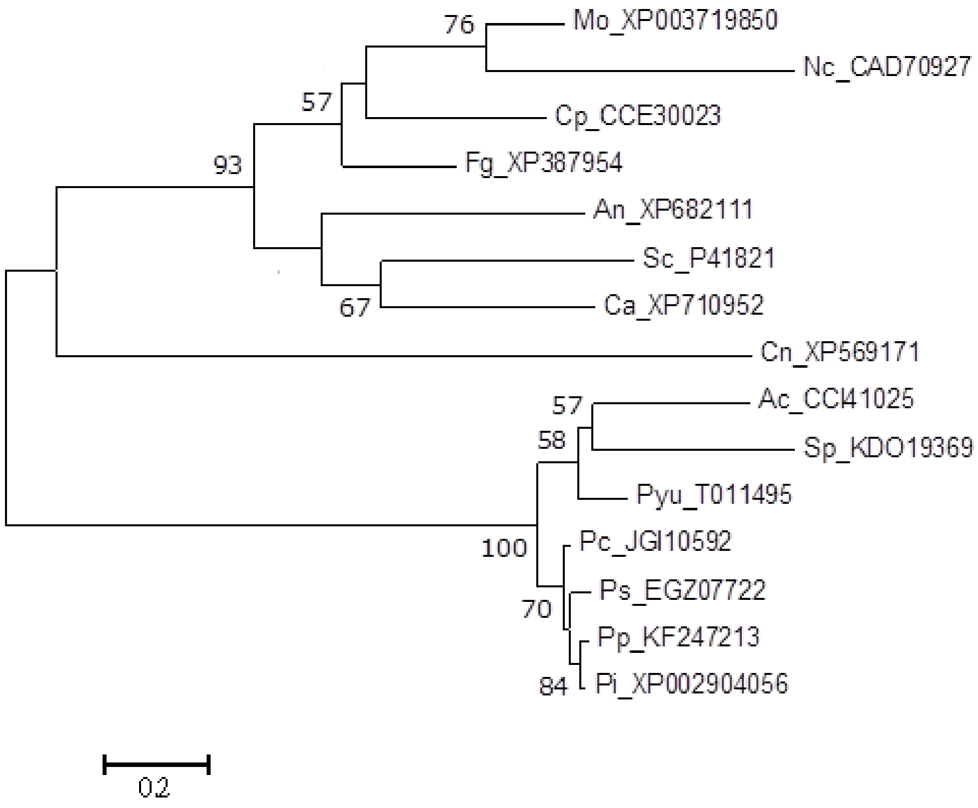


**Figure S2. Phylogeny analysis of *PpMID1* and its homologs from other organisms.** The phylogenetic tree was constructed by using the Neighbor-Joining algorithm implemented in MEGA 6.06 with 1,000 bootstrap replicates. Only bootstrap values > 50 are shown.
